# A new finite element based parameter to predict bone fracture

**DOI:** 10.1371/journal.pone.0225905

**Published:** 2019-12-05

**Authors:** Chiara Colombo, Flavia Libonati, Luca Rinaudo, Martina Bellazzi, Fabio Massimo Ulivieri, Laura Vergani

**Affiliations:** 1 Department of Mechanical Engineering, Politecnico di Milano, Milano, Italy; 2 TECHNOLOGIC S.r.l. Hologic Italia, Lungo Dora Voghera, Torino, Italy; 3 Fondazione IRCCS Cà Granda Ospedale Maggiore Policlinico, Nuclear Medicine-Bone Metabolic Unit, Milano, Italy; University of California Davis, UNITED STATES

## Abstract

Dual Energy X-Ray Absorptiometry (DXA) is currently the most widely adopted non-invasive clinical technique to assess bone mineral density and bone mineral content in human research and represents the primary tool for the diagnosis of osteoporosis. DXA measures areal bone mineral density, BMD, which does not account for the three-dimensional structure of the vertebrae and for the distribution of bone mass. The result is that longitudinal DXA can only predict about 70% of vertebral fractures. This study proposes a complementary tool, based on Finite Element (FE) models, to improve the DXA accuracy. Bone is simulated as elastic and inhomogeneous material, with stiffness distribution derived from DXA greyscale images of density. The numerical procedure simulates a compressive load on each vertebra to evaluate the local minimum principal strain values. From these values, both the local average and the maximum strains are computed over the cross sections and along the height of the analysed bone region, to provide a parameter, named Strain Index of Bone (*SIB*), which could be considered as a bone fragility index. The procedure is initially validated on 33 cylindrical trabecular bone samples obtained from porcine lumbar vertebrae, experimentally tested under static compressive loading. Comparing the experimental mechanical parameters with the *SIB*, we could find a higher correlation of the ultimate stress, σ_ULT_, with the *SIB* values (R^2^_adj_ = 0.63) than that observed with the conventional DXA-based clinical parameters, i.e. Bone Mineral Density, BMD (R^2^_adj_ = 0.34) and Trabecular Bone Score, TBS (R^2^_adj_ = -0.03). The paper finally presents a few case studies of numerical simulations carried out on human lumbar vertebrae. If our results are confirmed in prospective studies, *SIB* could be used—together with BMD and TBS—to improve the fracture risk assessment and support the clinical decision to assume specific drugs for metabolic bone diseases.

## 1. Introduction

Osteoporosis is a skeletal disorder that causes a reduction in bone strength and an increase in fracture risk [1]. It represents today a major public health issue, which affects more than 200 million people around the world, and in particular Caucasian women. The direct medical costs, associated with osteoporosis fractures, are particularly high (about 32 billion in Europe only) and are expected to increase owing to an aging population [2].

Therefore, extensive research has been promoted in establishing preventive and screening methodologies, as well as guidelines for drug treatments for people affected or subjects at risk [3]. Particular attention has been paid to improve the early diagnosis of osteoporosis of trabecular bones, where fracture risk due to osteoporosis is higher [4]. In particular, together with hip fractures, vertebral fractures are among the most common, leading to unfavorable and long-term consequences.

Bone strength is affected by two main factors: i) the three-dimensional structure of the bone, i.e. size, shape and distribution of bone mineral within the structure, partially captured by the 2D metric of areal bone mineral density, BMD; and ii) bone quality, where bone quality refers to architecture, turnover, damage accumulation (e.g. microfractures), and mineralization [5]. At present, areal bone mineral density (BMD) measured by DXA is one of the main clinical parameters for fracture risk assessment. However, BMD, which is a two-dimensional parameter, does not account for factors such as the three-dimensional microarchitecture, the damage accumulation, the turnover, and the quality of the bone organic phase, for instance related to the presence of Type 1 collagen. Indeed, BMD can predict only about 70% of vertebral fractures [3,6,7]. Several clinical studies show the effectiveness of textural-architectural parameters, such as Trabecular Bone Score (TBS) in improving the prediction of bone fractures [8,9], by providing a quantitative measurement of the bone quality (referred to the skeletal architecture) not obtained by BMD. TBS can predict fragility fractures also independently from BMD in retrospective and longitudinal studies [10]. Moreover, patients affected by secondary osteoporosis, like due to diabetes, suffer from fragility fractures with normal or slightly reduced BMD, but may present low TBS [11].

However, there is still about a 30% of unpredicted fractures, which could be explained by the impairment of bone “quality” parameters, e.g. geometric and material factors contributing to fracture resistance independently of bone mineral density [7,12,13]. Partially, TBS can help BMD decreasing this percentage of unpredicted failures [14–16], but some further improvements can be gained with specific parameters.

Other techniques, alternatives to DXA, such as quantitative computed tomography (qCT) applied for the assessment of hip fractures [17] and of vertebral fractures of men in vivo [18], or as digital tomosynthesis applied in [19] for the assessment of spine fractures, are rarely applied in the clinical routine due to the high radiation required and limited availability. Finite element (FE) models, derived from qCT-scans, were also developed as a clinical tool to evaluate vertebral strength, showing a good correlation in the prediction of the experimental vertebral strength [20]; similarly, for digital tomosynthesis, the work [21] compared strains from linear 3D μ-FE models with experimental strain distributions from Digital Volume Correlation measurements. Although these techniques provided an alternative clinical tool to DXA for osteoporotic patients, their use for routine diagnosis was limited due to the high dose, time, and cost of qCT. To overcome these limitations, Choisne [22] recently proposes a novel approach based on bi-planar DXA to build vertebral FE models from synchronized sagittal and frontal plane radiographs.

The results are encouraging and showed that the vertebral strength, determined from FE models, is a strong predictor of the experimental failure load. This method allows for fast, low-radiation, and minimal cost patient-specific 3D FE model as accurate as qCT-based or digital tomosynthesis based FE models.

Although FE models based on bi-planar DXA could be a good alternative to replace qCT-based models in the prediction of vertebral strength, they still require a certain dose of radiation and an additional procedure.

Micro-finite element models based on micro-computed tomography (micro-CT) images play a fundamental role in studying and understanding bone failure processes and bone remodeling [23–25], however this technique is not currently applied for clinical measurements because images are obtained by scanning ex-vivo specimens.

Literature reports some DXA-based finite element models to evaluate a fracture risk index, but mainly focused on hip fracture [26–28]. In particular, Naylor et al. [26] propose a DXA-based FE model tool for the assessment of bone strength, able to identify patients at high risk of hip fracture who may benefit from treatment to reduce fracture risk. Mancuso et al. [27] show that higher levels of adult physical activity, grip strength, and body mass result in a favorable bone microstructure structure and lower numerical strains. Moreover, they also show that areal BMD does not capture the wide range of strains experienced during typical physiologic loading. Moreover, they also show that areal BMD does not capture the wide range of strains experienced during typical physiologic loading. Yang et al. [28] propose an approach based on the 2D DXA images of femur; these authors adopt a mesh with triangular elements and assign homogeneous properties to the tissue.

In the present study, we propose a novel numerical approach based on the Finite Element Method (FEM), derived from DXA greyscale images of density distribution measured on trabecular bone tissue from porcine lumbar vertebrae. The numerical procedure simulates a compressive load on each vertebra to evaluate the local strain values. The greyscale-based mesh and local properties are fundamental and aim to feed the model with additional information, when compared to [28], regarding the local stiffness distribution of the bone. We believe that providing a local distribution of mechanical properties enriches the numerical model, provides a better representation of the reality and ensures a better estimation of fracture, especially considering that it is a local phenomenon. From these values, a novel parameter, named Strain Index of Bone (*SIB*), is developed and correlated with bone strength. The procedure is first validated on cylindrical trabecular bone samples obtained from porcine vertebrae, experimentally tested under static compressive loading. Finally, we present a few numerical case studies carried out on human lumbar vertebrae. The proposed procedure could represent a complementary numerical tool, based on a patient-specific FE model, to improve the DXA accuracy. The proposed FE-strain based parameter, *SIB*, incorporates information on the geometry, density distribution from DXA measurements, and loadings and is defined as a local quantity, differently from BMD and TBS, which are averaged over the scanned region. This choice is based on previous literature findings [29–31] that showed that failures occurred in local bands and how local discontinuities, porosities, and microstructure could be considered as a source of damage initiation.

## 2. Materials and methods

The proposed numerical procedure is applied to porcine samples for a first validation and then to the human vertebrae. This study reports anecdotal examples for a new numerical procedure analysis of bone quality applied to lumbar DXA scans of three patients (Approval of the Local Ethical Committee: Comitato Etico Milano Area 2. Protocol N 2.0 BQ. 265_2017, 13th June 2017). All the three patients provided their written informed consent.

### 2.1 Porcine bone samples

Porcine samples were experimentally tested by a combination of characterization techniques and then were analysed by the proposed numerical procedure.

#### 2.1.1 Experimental procedure

**Fig 1:**
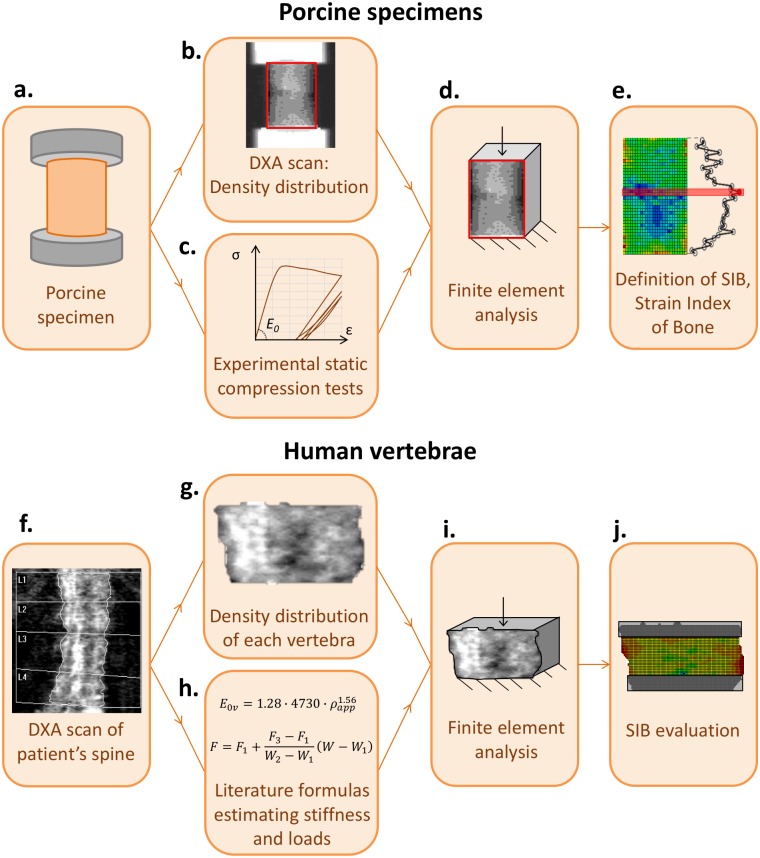
Scheme of the adopted procedures for porcine bone specimens (a. to e.) and for human vertebrae (f. to j.). Porcine bone specimens: a) Cylindrical sample of trabecular bone obtained from porcine vertebrae and embedded into aluminium endcaps. b) Greyscale 2D-image of density distribution obtained from DXA scanning. c) Experimental compressive testing on bone samples. d) FE-analyses on simplified sample geometry and DXA-based stiffness distribution. e) Definition of the Strain Index of Bone (*SIB*) based on the outcome of the numerical analyses. Human vertebrae: f) DXA scan of the spine, evidenced with a white border, 1 pixel thick. g) Greyscale 2D-image of the density distribution of a single vertebra, obtained from DXA scanning. h) Selection of literature formulas to estimate stiffness and loads from the density. i) FE-analyses on simplified sample geometry and DXA-based stiffness distribution. e) estimation of *SIB* based on the outcome of the numerical analyses.

The experimental procedure is described in detail in a previous work [32] and includes the following steps (Fig 1a–1c):

1. **Sample preparation**: 40 cylindrical trabecular bone specimens were cored from six different porcine vertebral columns. The ends of bone samples were glued in aluminium endcaps. The nominal diameter of the cylindrical samples was 13.8 mm and the nominal height 30 mm. However, for the following analyses and as in [32], the duplicates, i.e. specimens extracted from the same lumbar vertebra, were removed. This leads to 33 analysed specimens;
2. **Analysis of undamaged samples**: DXA images were collected adding 20mm layer of solid water provided by Gammex on undamaged samples, by using a Hologic Discovery A system (Hologic Inc, Marlborough, Massachusetts, USA) installed at the Bone Metabolic Unit of the Nuclear Medicine of the Fondazione IRCCS Ca’ Granda-Ospedale Maggiore Policlinico, Milan, Italy. Hologic densitometers are dual-energy pulsed systems with pulsed kV between 100 and 140kV. Image segmentation is performed automatically by SW Apex 3.3 installed on Hologic Discovery^™^. The collected images are projections of the scans anteroposterior, with a DXA resolution of 0.5mm. They were collected on the cylindrical trabecular samples between the endcaps. The purpose of these pre-damage DXA measurements, and the related numerical models described in the next sub-section, is to estimate the failure cross-sections and to quantify damage propensity of the specimens;
3. **Mechanical testing**: monotonic compressive tests were performed in displacement control (strain rate of 0.0002 s^-1^; constant stroke rate of 0.05 mm/s). Three preconditioning compression cycles up to 0.1% axial strain were performed, followed by monotonic loading until certain strain levels. After that, the specimens were unloaded and loaded three times to the same strain level, to obtain mechanical damage. Samples were divided into four groups, each set loaded until reaching a specific engineering strain value:
  - Group *G1%*, with specimens loaded until 1% strain;
  - Group *G2%*, with specimens loaded until 2% strain;
  - Group *G3*.*5%*, with specimens loaded until 3.5% strain;
  - Group *G5%*, with specimens loaded until 5% strain.
  From now on, we will also refer to these groups as ‘strain levels’ or ‘damage levels’.
4. **Analysis of damaged samples**: DXA scanning was performed in air on damaged samples (i.e. after performing the experimental tests). The purpose of these post-damage DXA measurements and the related numerical models is to verify the estimations from the pre-damage DXA and models and to quantify the damage level induced by given mechanical loading.

More details about the experimental testing protocol are provided as **Supporting Information**.

#### 2.1.2 Numerical procedure

**Fig 2:**
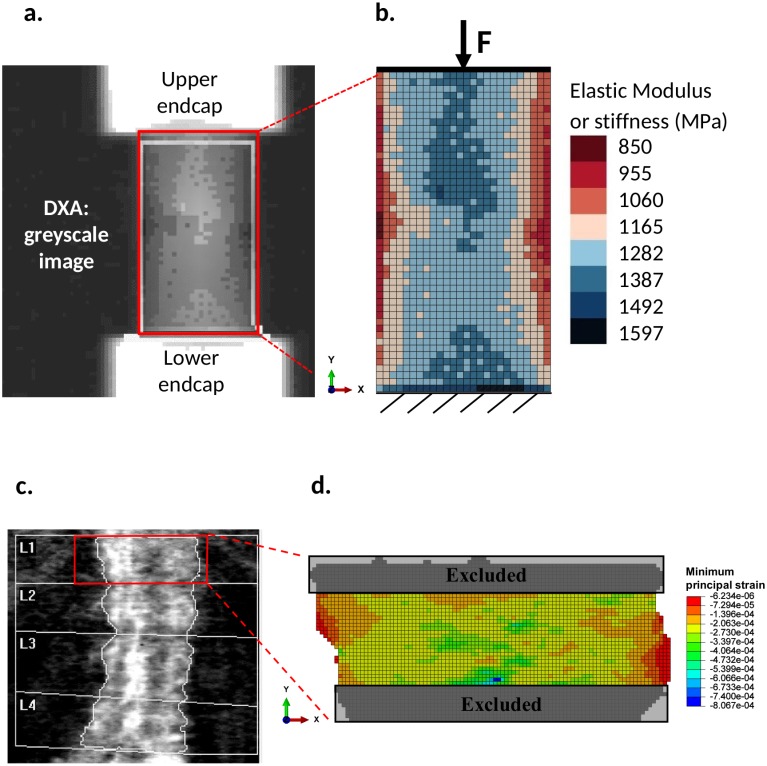
a) Greyscale image obtained from a DXA on a porcine bone sample. b) Schematic of the FE-model of the cylindrical sample, including the mesh, the loading and boundary conditions. Each colour corresponds to a specific stiffness value, as shown in the colour bar. c) Greyscale image obtained from a DXA of a spine from a patient fractured not in the lumbar area (corresponding to P3 in Table 2). b) Example of minimum principal strain (*ε*_*pmin*_) field in vertebra L1.

The Finite Element numerical tool used for the simulations is a commercial software, ABAQUS (v. 2017, SimuliaTM, Dassault Systèmes^®^). The numerical procedure includes the following steps:

1. **Model geometry**. The numerical model is a simplification of the real 3D cylindrical geometry of the sample. We created a 2D model with a rectangular geometry based on the greyscale matrix of the density, obtained from the DXA scanning (Fig 1d). To build a 2D plane strain FE model, we accounted for a fictitious constant thickness t*, evaluated from the equivalence between the cross-section of the cylindrical real specimen, *A*_*s*_, and the cross-section of the 2D-FE model, *A*_*FEM*_:
  $$\left\{ \begin{array}{l} A_{s}=\pi r^{2}\\ A_{FEM}=t^{*}\cdot 2r \end{array}\right.$$ (1)
  where *r* is the radius of the cylindrical specimen, experimentally measured on each sample. Assuming that the two cross-sections are equivalent (*A*_*s*_ = *A*_*FEM*_), we obtained the equivalent thickness t* of the 2D-FE model as in Eq (2):
  $$t^{*}=\frac{\pi r}{2}$$ (2)
  The geometrical model is then discretized into square elements corresponding to the pixels of the DXA image. The DXA output image is imported in Matlab^®^ (v. R2018a, The MathWorks, Inc.) as a matrix, whose entries are numbers that indicate the brightness of the pixel (i.e. 0–255 code number, where 0 stands for black colour and absence of bone, and 255 stands for white colour and maximum bone density). Each element of the matrix, corresponding to a pixel of the DXA image, represents a square element of the mesh. This allows one working with a limited number of elements, with a constant size equal to the DXA pixel resolution (500x500 μm). The used elements are 2D continuum plane strain element with four integration points (type CPE4 in ABAQUS).
2. **Material characteristics and boundary conditions**. The material behaviour is linear elastic. The implemented numerical procedure is not aimed at simulating the damage. Rather the focus is on the correlation between parameters estimated before damage onset and the effective quantities experimentally determined at the beginning of the damage (yielding stress and strain) and at failure (ultimate stress and strain). The choice of a linear elastic model is also motivated by the possibility of reducing the computational time, enabling easy implementation of the numerical tool into the computer used for the DXA in clinical routine. To assign a local value of elastic modulus to each element, we considered a linear proportion between the values of greyscale, *G*_*i*_, and the values of local stiffness, *E*_*i*_, by associating the value of global stiffness, i.e. the elastic modulus *E*_*0*_ experimentally measured from the compressive experimental tests [32], with the mean grey value $\bar{G}$:
  $$\bar{G}=\frac{\sum_{i=1}^{N}G_{i}}{N}$$ (3)
  $$E_{i}=\frac{E_{0}\cdot G_{i}}{\bar{G}}$$ (4)
  where:
  - $\bar{G}$ is the mean greyscale value of the scanned DXA matrix, defined in Eq (3);
  - *G*_*i*_ is the greyscale level corresponding to *i-th* pixel;
  - *N* is the total number of pixels of the scanned DXA matrix, which corresponds to the total number of elements of the 2D model;
  - *i* is the index scanning rows and columns of the greyscale matrix;
  - *E*_*i*_ is the elastic modulus of the *i-th* element, defined in Eq (4);
  - *E*_*0*_ is the initial global elastic modulus measured from the experimental compressive test.
  With this simple procedure, we can obtain a matrix containing the scaled local values of stiffness, *E*_*i*_, according to *E*_*0*_ and $\overset{-}{G}$. The Poisson ratio is set constant and equal to 0.3. We assigned the same stiffness, *E*_*i*_, to elements corresponding to pixels with the same greyscale value. Fig 2 shows an example of a DXA scan (Fig 2a) and the corresponding FE-model (Fig 2b), where the colour bar indicates the different local elastic moduli assigned to the bone tissue. This procedure allows all the information, obtained from the DXA image (i.e. 2D specimen dimension, distribution of the bone mass, and material properties of each pixel or element), to be included in the FE-model.
  Boundary conditions are applied to reproduce the uniaxial compressive tests (Fig 2b), by constraining all the degrees of freedom at the bottom edge and applying a vertical force, F, uniformly distributed to the upper edge. To apply a uniformly distributed force, we used a kinematic coupling. We applied the same load value, F = 60N, to all the samples, by considering that all the animals have similar age and weight. The value of the applied load is selected to obtain stresses and strains of the same magnitude order of the values determined for humans in standing position.
3. **Numerical analyses and data post-processing**. Linear elastic analyses, aimed at simulating the uniaxial compressive tests, were performed on each model corresponding to a bone sample. From the displacements of the nodes, a numerical elastic modulus, *E*_*FEM*_ is calculated, for each sample, by Eq (5):
  EFEM=FAFEMuyh=F(2rt*)uyh=Fuy⋅h2rt* (5)
  where:
  - *F* [N] is the applied compressive force, equal to 60 N;
  - *A*_*FEM*_ [mm] is the rectangular cross section of the equivalent 2D-FE model (*A*_*FEM*_ = 2*r* ∙ t*);
  - t* [mm] is the equivalent thickness;
  - *h* [mm] is the height of the model;
  - *u*_*y*_ [mm] is the vertical displacement of the upper surface of sample obtained by numerical analyses.

From the strain values we defined *SIB* as the minimum principal strain, ε_pmin_, (maximum in modulus) calculated at each integration point. For each *j-th* element, having four integration points, we calculated the mean, *SIB*_*mean*,*j*_, and maximum (in modulus), *SIB*_*max*,*j*_. The mean and the maximum value over the sample are calculated using Eqs (6) and (7), respectively:

$$SIB_{mean}=\frac{\sum_{k=1}^{4N}\left|\varepsilon_{p\text{min},k}\right|}{4N}\cdot 10^{4}$$ (6)

$$SIB_{\text{max}}=\text{max}\left|\varepsilon_{p\text{min}}\cdot 10^{4}\right|$$ (7)

where *k* is the variable scanning the integration points of the whole model, ranging from 1 to 4*N*, and *N* is the total number of elements in each model, which corresponds to the total number of pixels in the DXA image. 10^4^ is a multiplicative factor to obtain more readable values. The *SIB* values are calculated, for each sample, considering the scanned bone region and excluding 5 rows of pixels, at the upper and lower regions, i.e. 2.5mm per side, where the strain field could be affected by the applied boundary conditions. An example of the *SIB* trend along the specimen height is given in Fig 1e, where the red line highlights the specimen cross section with the highest *SIB*.

Besides, we also numerically calculated the values of the Strain Index of Bone in correspondence of the yielding strain, *SIB*_*Y*_. *SIB*_*Y*_ values are obtained applying to each model the specific experimental yield force, *F*_*Y*_, evaluated at the linear-elastic limit of the stress-strain curve obtained from the corresponding sample.

We performed a statistical analysis to estimate the correlations between the numerical results (*SIB*_*max*_ and *SIB*_*mean*_), and the experimental clinical (BMD and TBS) and mechanical (initial elastic modulus E_0_, yield strain ε_Y_ and ultimate strain at failure ε_UTS_, and yield stress σ_Y_ and ultimate strength σ_UTS_) parameters measured on the same batch of porcine samples and presented in [32]. The software used for the statistical data analysis is Matlab^®^ (v. R2018a, The MathWorks, Inc.), and specifically, the function *fitlm*, fitting a linear model and calculating the main statistical parameters. Correlations are estimated based on the ordinary (unadjusted) determination coefficient R^2^ and the adjusted determination coefficient R^2^_adj_, defined in [33,34].

### 2.2 Human vertebrae: Case studies

We propose the application of a numerical procedure, based on experimental DXA scanning, to human vertebrae, similarly to the porcine specimens described in the previous section. The validation of this procedure can be performed based on a wider experimental campaign with statistical evidence, which is beyond the aim of this work. The case studies we present will allow discussing the methodology and the potential applicability in the clinical routine.

#### 2.2.1 Experimental procedure

Three spines, belonging to i) a non-fractured patient, ii) a lumbar-fractured patient, and iii) a non-lumbar fractured patient, i.e. a patient with osteopenia who presented with an osteoporotic fracture at another site than the lumbar spine, were scanned by the same DXA machine, Hologic Discovery A system, adopted for the porcine bone specimens. The scans were performed at the Bone Metabolic Unit of the Nuclear Medicine of the Fondazione IRCCS Ca’ Granda-Ospedale Maggiore Policlinico, Milan, Italy. The DXA images were collected *in-vivo* during the clinical routine controls. The patients are elderly women, 73±9 y.o., with different health conditions. Fig 1f shows an example of one of these scanned spines, with the greyscale output image. Fig 1g gives details of one vertebra.

#### 2.2.2 Numerical procedure

This procedure includes the following steps:

1. **Model geometry**. Human lumbar vertebrae have different complex morphologies and load capacities as a function of their anatomical position. We assumed that the vertebrae are similar to cylinders, thus excluding the vertebral arc and focusing the attention only on the vertebral body, mainly made of trabecular tissue. Even if this hypothesis can limit the precision of the suggested model, we adapted the procedure used for porcine samples to human vertebrae, to have a rapid and easy tool to be implemented in the post-processing of the DXA measurements. The thickness of the vertebral body, t*, is calculated from the width of each cross section of the vertebra. Assuming a cylindrical geometry of the vertebra, as in [35] the average width represents the mean vertebral diameter. Thus, we can calculate t* or the vertebra as from Eq 2:
  $$t^{*}=\frac{\frac{\pi }{2}\frac{\sum_{j=1}^{n}w\left(j\right)}{n}}{2}$$ (8)
  where:
  - *j* is the variable scanning the rows of the greyscale matrix of the processed region of the vertebra;
  - *w(j)* is the width in correspondence of the j-th cross section;
  - $\frac{\sum_{j=1}^{n}w\left(j\right)}{n}$ is the average width, corresponding to the average diameter.
  Then, in Eq (9) we converted the areal bone mineral density (BMD) into a volumetric bone mineral density (vBMD), based on t*, as suggested by Yang et al. [36]:
  $$vBMD=\frac{BMD}{t^{*}}$$ (9)
  From the values of vBMD, we estimated by Eq (10) the apparent density, *ρ*_*app*_, as in [37]:
  $$\rho_{app}=\frac{vBMD}{\left(1.14\cdot 0.598\right)}$$ (10)
2. **Material characteristics and boundary conditions**. We derived the stiffness *E*_*0v*_ of each vertebra from Eq (11), which is specific for human bones with a standard error of prediction of 23%, according to previous studies [38–40]:
  $$E_{0v}=1.28\cdot 4730\cdot \rho_{app}^{1.56},\;\text{for}\;\rho_{app}<0.35\frac{g}{cm^{3}}$$ (11)
  To assign a specific elastic modulus to each pixel, according to the greyscale level of the DXA image (Fig 2c), we adopted a similar procedure to the one used for the porcine specimens. We implemented a linear proportion between the elastic modulus, calculated in Eq (11), the mean values of the greyscale $\bar{G}$, calculated as in Eq (3), and the considered pixel, similarly as in Eq (4). The Matlab code, implemented for porcine specimens, was modified and adapted to the case of human vertebrae. Particular attention was paid to the automatic selection of the vertebral area from the DXA output images. The DXA scanning machine evidences automatically the vertebra flanks with white borders, one pixel thick. This is done on the image of the whole spine, which is then cropped. Then, borderlines are separately saved in a binary matrix. In particular, the irregularity of the sides is automatically selected masking the background with specific Matlab commands (i.e. *bwselect* to create the mask and *imshowpair* to compare the differences between the original DXA image and the mask). The boundary conditions are the same adopted for the cylindrical samples: a compressive force is applied at a point vertically aligned with the centroid of each vertebral area and located in correspondence of the highest ordinate of the vertebra, and all the degrees of freedom of the bottom edge are constrained. For the loading and boundary conditions, we considered only the elements in common between the studied vertebra and the upper or lower ones, i.e. the intervertebral disk is not considered. This loading case wants to simulate the standing position of the patient. Of course, this is only one of the possible loading modes of the spine, since bending and torsion can occur as well. The proposed numerical model represents a first attempt to simulate the mechanical behaviour of the human and, owing to its two-dimensional nature, we limited the simulations to the simple uniaxial compressive loading.
  The applied load, *F*, is specific for each patient and each vertebra of the spine. The calculation of *F* is based on the work by Han et al. [41], which analysed women with weight 50–120 kg and height from 150–200 cm, covering 99% of the world population. According to [41,42], the load acting on the lumbar vertebrae in the standing posture is approximately linearly dependant on the variation of body weight, rather than on the body height itself. Thus, the value of *F* is estimated by interpolating the values in [41] as in Eq (12):
  $$F=F_{1}+\frac{F_{3}-F_{1}}{W_{2}-W_{1}}$$ (12)
  where:
  - *W* [kg] is the patient’s total body weight;
  - *W*_*1*_ and *W*_*2*_ [kg] are the reference extreme weights, respectively of 50 and 120;
  - *F*_*1*_ [N] is the resultant force at the considered lumbar vertebra for a person of 50 kg and 150 cm, according to [41];
  - *F*_*3*_ [N] is the resultant force at the considered lumbar vertebra for a person of 120 kg and 150 cm according to [41].
3. **Numerical analyses and data post-processing**. Linear elastic analyses, aimed at simulating the uniaxial compression were performed on each model. Similarly to the case of the porcine specimens, we excluded from the post-processing of numerical outputs 10 rows of elements (corresponding to 5mm) from both the upper and from the lower regions (Fig 2d), owing to the strain concentration caused by the boundary conditions. From the FE-simulations, we obtained the strain field and the *SIB*. In particular, *SIB*_*mean*_ and *SIB*_*max*_, using Eqs (6) and (7), respectively. The results were processed considering each vertebra, row by row.

## 3. Results

### 3.1 Porcine bone samples

**Fig 3:**
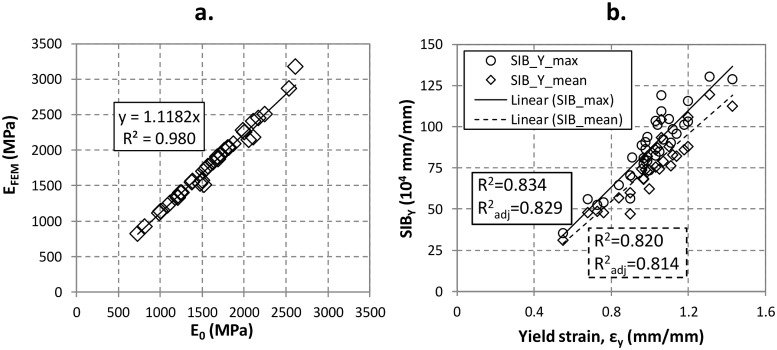
Validation of the numerical procedure: a) Linear correlation between the numerical global stiffness, *E*_*FEM*_, and the experimental global stiffness, *E*_*0*_. b) Linear correlation between the numerical Yield Strain Index of Bone *SIB*_Y_, the and experimental yield strain, *ε*_*Y*_. The linear correlation is shown for both the maximum *SIB*_Y_ value, *SIB*_*Ymax*_, and the mean *SIB*_Y_ value, *SIB*_*Ymean*_. *SIB*_*Y*_ is calculated, for each model, by using the load causing yield in each sample.

Fig 3a shows the trend of the modulus of elasticity numerically calculated, *E*_*FEM*_, *v*ersus the values experimentally determined, *E*_*0*_, for each sample. Fig 3b shows the experimental values of the yield strain, *ε*_*Y*_, versus the numerical Strain Index of Bone calculated in correspondence of the yielding strain, (*SIB*_*Y*_). The yield strain was experimentally measured during the tests as the linear-elastic limit of the stress-strain curve, whereas the *SIB*_*Y*_ values are obtained numerically.

The significant linear correlations (R^2^ = 0.980) between the numerical and the experimental values of elastic modulus and for both the maximum *SIB*_*Y*_ value, *SIB*_*Ymax*_, and the mean *SIB*_Y_ value, *SIB*_*Ymean*_, with R^2^ = 0.834 and R^2^ = 0.820, respectively, validate the adopted numerical framework, also confirming the validity of the weighting procedure used to assign the local elastic moduli to each element of the model. This means that the distribution of the local stiffness according to the linear proportion between the elastic modulus and the local bone mineral density given in Eq (4) is representative of the real one.

**Fig 4:**
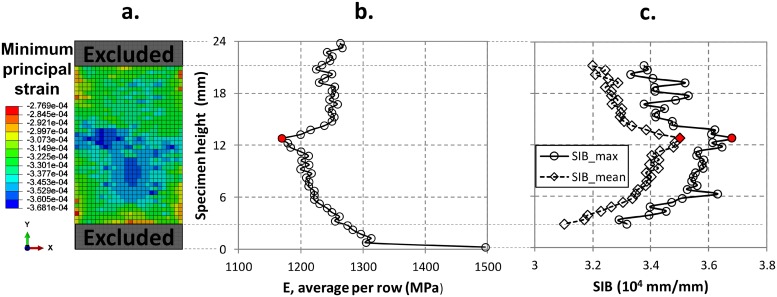
Results of the numerical simulations referred to the specimen in Fig 2: a) Minimum principal strain field (*ε*_*pmin*_). The grey-coloured regions at the upper and lower grips are excluded from the *SIB* post-processing. b) Trend of the average local stiffness *E* per rows through the specimen height. The red dot evidences the section with minimum stiffness. The distance along y-axis is the measure from the bottom grip (0 mm) to the upper grip (24 mm). c) Trend of *SIB*_*max*_ and *SIB*_*mean*_ per rows along the specimen height. The red dot evidences the section with maximum *SIB*.

The strain field is post-processed by computing the average stiffness for each row of elements, to identify the weakest section of each specimen. Fig 4a shows an example of the distribution of the minimum principal strain in the same sample model of Fig 2. Fig 4b and 4c show the values of the estimated elastic modulus, *E*, and the trends of *SIB*_*max*_ and *SIB*_*mean*_ per row. The plots in Fig 4b and 4c identify a section where the average stiffness per row is minimum and the corresponding strains are maximum. Since the model is linear elastic and the applied load is the same for all the specimens, there is a net correlation between the minimum value of *E*, corresponding to the maximum compliance, and the maximum values of *SIB*.

Table 1 summarizes all the adjusted linear determination coefficients among the main clinical parameters (*i*.*e*. BMD and TBS), the mechanical properties (*i*.*e*. *E*_0_, *ε*_*Y*_, *σ*_*Y*_, *ε*_*ULT*_, *σ*_*ULT*_), and the numerical quantities (*i*.*e*. *SIB*). We can consider these determination coefficients as meaningful when the p-value is smaller than 0.05, as given in Table 1. To compute these correlations, we considered all the available specimens, by pooling the data from all the samples belonging to different damage groups. Given the results of Table 1, we can evidence that the two *SIB* parameters have similar determination coefficients. We will focus on *SIB*_*max*_ in the following discussion, because the authors suppose that the damage is a local phenomenon, according to [30,31].

**Table 1:** Adjusted linear determination coefficients ($R_{adj}^{2}$) between the numerical *SIB*_*max*_ and *SIB*_*mean*_, and the experimental clinical and mechanical parameters. Grey-coloured cells represent a significant linear correlation, with *p-value* < 0.05.

	Experimental clinical parameters [32]		Experimental mechanical parameters [32]					Numerical mechanical parameters	
	**BMD**	**TBS**	***E***_***0***_	***ε***_***Y***_	***σ***_***Y***_	***ε***_***ULT***_	***σ***_***ULT***_	***SIB***_***max***_	***SIB***_***mean***_
**BMD**	1	0.03	**0.2**	-0.03	**0.23**	-0.03	**0.34**	**0.26**	**0.24**
**TBS**		1	-0.03	**0.13**	**0.10**	-0.04	-0.03	-0.03	-0.03
**E**_**0**_			1	**0.18**	**0.42**	**0.16**	**0.67**	**0.86**	**0.86**
**ε**_**Y**_				1	0.08	0.01	-0.01	**0.20**	**0.19**
**σ**_**Y**_					1	0.08	**0.76**	**0.35**	**0.36**
**ε**_**ULT**_						1	-0.04	**0.25**	**0.25**
**σ**_**ULT**_							1	**0.63**	**0.65**
***SIB***_***max***_								1	**1.00**
***SIB***_***mean***_									1

**Fig 5:**
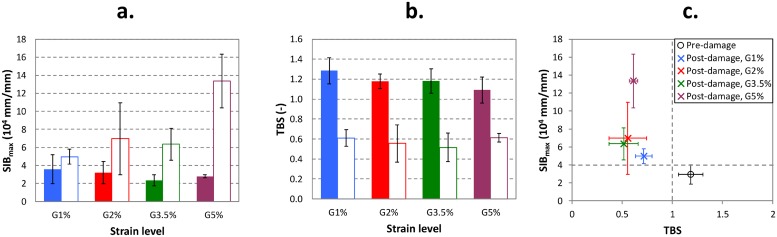
Variation of: a) *SIB*_*max*_ and b) TBS—before and after damage—for different mechanical damage levels (*G1%*, *G2%*, *G3*.*5%*, and *G5%*). The filled bars represent the results before damage and the unfilled ones after damage. Panel (b) reproduced with permission from (Mirzaali et al., 2018) PLOS ONE 2018. c) Trend of *SIB*_*max*_ vs TBS for pre- and post-damaged specimens considering the mean value and with the standard deviation. Each colour corresponds to a different damage level.

Fig 5 shows the trend of *SIB*_*max*_ for the pre- and post-damaged specimens, divided for each strain group (i.e. *G1%*, *G2% G3*.*5%* and *G5%*), compared with the TBS trends. The variation of *SIB*_*max*_ between pre- and post-damage is approximately 39%, for *G1%*, and up to 380%, for *G5%*. *G1%* is the only group where the pre- and post-damage confidence bands are very close. For all the other groups we can notice a clear difference, more pronounced in the case of G5%. The **Supporting Information** reports the same plot of Fig 5a for *SIB*_*mean*_, showing a similar trend.

By comparing the analyses performed on pre and post-damaged samples, we noticed that the sections characterized by the highest values of *SIB*_*max*_ are the same in pre- and post-damaged samples. More specifically, we found that the row where *SIB*_*max*_ is maximum (i.e. the cross-section distance from the bottom grip) was equal or very close (± 1mm) between pre- and post-damaged specimens.

Fig 5c provides a direct comparison between TBS and *SIB*_*max*_. There is a net separation between pre- and post-damaged samples in terms of TBS, which could be represented by a vertical line at TBS = 1. On the other hand, it is possible to sketch a horizontal line, even if less evident due to the higher scatter of some post-damaged groups, that could state the presence/absence of damage at about *SIB*_*max*_ = 4.

### 3.2 Human vertebrae

Table 2 shows a comparison between the conventional clinical parameters (BMD, T-score, and TBS) and the proposed one, *SIB*_*max*_, for three patients, including fractured and non-fractured.

**Table 2:** Summary of BMD (g/cm^2^), T-score, TBS and *SIB*_*max*_ (10^4^ mm/mm) for each vertebra of three case-studies on patients.

	P1, non-fractured				P2, lumbar fractured				P3, non-lumbar fractured			
Lumbar vertebra	BMD	T-score	TBS	*SIB*_*max*_	BMD	T-score	TBS	*SIB*_*max*_	BMD	T-score	TBS	*SIB*_*max*_
**L1**	1.454	+4.8	1.436	1.230	0.634	-3.2	1.163	10.317	0.838	-1.4	0.965	8.356
**L2**	1.310	+2.6	1.473	2.738	n.a.	n.a.	n.a.	n.a.	0.819	-1.9	1.002	4.466
**L3**	1.488	+3.7	1.492	1.474	0.635	-4.1	1.334	4.410	0.848	-2.1	1.096	5.259
**L4**	1.453	+3.1	1.364	2.447	0.706	-3.2	1.170	5.322	0.837	-2.0	1.058	4.012
**Average value**	1.430*	+3.5*	1.441	1.972	0.661*	-3.6*	1.222	6.683	0.836*	-1.9*	1.030	5.523

* BMD and T-score average values are calculated with a proprietary formulation of the DXA scanning machine.

## 4. Discussion

This study proposes a new parameter, the Strain Index of Bone, the aim being to improve the ability of DXA-based clinical analyses, in predicting the strength and fragility of trabecular bones. The adopted numerical procedure is first validated on the experimental testing carried out on *ex-vivo* samples taken from porcine vertebrae. Then, it is used to compare the *SIB*_*max*_ with the clinical and mechanical parameters and to investigate how the *SIB*_*max*_ is influenced by mechanically-induced damage.

The determined correlations among clinical, experimental and numerical mechanical parameters (Table 1) evidence that *SIB*_*mean*_ and *SIB*_*max*_ are the best predictors of the compressive strength of trabecular bones, *σ*_*ULT*_, with determination coefficients, R^2^_adj_ = 0.65 for *SIB*_*mean*_ and R^2^_adj_ = 0.63 for *SIB*_*max*_, much higher than the correlation of BMD (R^2^_adj_ = 0.34) and that of TBS, which has a non-meaningful correlation (R^2^_adj_ = -0.03). *SIB* values are correlated with BMD (R^2^_adj_ = 0.26 for *SIB*_*mean*_ and 0.24 for *SIB*_*max*_), but are not correlated with TBS. For the sake of precision, we should mention that ultimate stress, σ_ULT_, and ultimate strain, ε_ULT_, were not available for all the considered specimens, because only specimens belonging to the groups *G3*.*5%* and *G5%* (17 specimens in total) reached the ultimate strength and strain, while the elastic modulus and the yielding strain and stress are calculated also from specimens of groups *G1%* and *G2%*.

Moreover, the outcome of this study suggests that *SIB* is a better predictor of the experimental vertebral strength than BMD and TBS, showing a two-fold increase in the correlation coefficient with respect to BMD. This outcome is in agreement with the recent results of [43]; these authors found that 2D DXA-based numerical models of human spines, compressed with a constant displacement, are able to estimate a load well-correlated with the experimental bone strength (R^2^ = 0.66), higher than the single BMD estimation (R^2^ = 0.56).

This improvement in the estimation of bone strength could be clinically relevant, with potential improvements in the early diagnosis of osteopenia, as the work [44] did by an experimental campaign, even if with a less detailed 2D model. The yielding properties (ε_Y_ and σ_Y_) showed lower correlation coefficients with *SIB*_*max*_ than the corresponding ultimate properties (ε_UTS_ and σ_UTS_). Bone is a rather complex material, and failure of the trabecular structure normally occurs when the ultimate tensile strength is reached. In all the samples analysed, the highest values of *SIB*_*max*_ were found in the regions characterized by the lowest stiffness, thus validating the ability of the proposed parameter in predicting, not only the strength but also the most critical zones of trabecular tissue. *SIB*_*max*_ has been shown to be largely sensitive to the damage and that it increases with the damage level. TBS, instead, is affected by the damage and always shows a post-damage reduction, but it is not sensitive to the damage level. Indeed, a large difference in applied strain levels does not correspond to a proportional variation of TBS value. TBS has shown to be a good indicator of the bone quality and of the presence of damage, without being able to quantify it though [45–50]. Also, it cannot be used to accurately explain the risk of fracture in the bone tissue or to define how healthy bone is, as it does not show a linear relationship with the mechanical strength [32]. The comparison between *SIB*_*max*_ and TBS shows how the latter is able to clearly distinguish between damaged and undamaged samples. The ability of *SIB*_*max*_, in assessing the presence of damage is a little less evident. However, *SIB*_*max*_ has shown to be more sensitive to the amount of damage (i.e. strain level), also if compared with other parameters such as BV/TV, BS/TV, Tb.Sp., Tb.Th. and DA, analysed in our previous study [32]. This means *SIB*_*max*_ has the potential to be a more quantitative parameter. TBS is directly related to the number of trabeculae and their connectivity and inversely related to the trabecular spacing. However, the number of trabeculae and their connectivity is not always an indicator of bone strength. The ability to withstand the load also depends on the local orientation of the trabeculae, which directly affects the local stiffness and strength. The skeletal architecture is paramount, as local stress concentration may arise, being caused by the trabecular thickness and orientation, and leading to premature failure, independently on bone mass.

This study introduces the *SIB* and supports its validity in damage identification and quantification when applied to the case of *ex-vivo* trabecular bone samples, from similar porcine spines and having low scattering in the mechanical properties, because pigs were of close age, weight and were all healthy. *SIB* from undamaged samples offers information about the weakest section, where damage is more prone to occur, i.e. failure propensity of bone; this identification is confirmed by the simulations on post-damaged specimens.

However, one should also point out some limitations of the procedure. The main one consists in reducing a 3D problem to a 2D model. This is due, basically, to the nature of 2D DXA images. As a consequence, the intrinsic thickness variability results in a pattern with a denser region at the core of the sample and less dense regions at the lateral edges (Fig 2b). Nevertheless, this simplified model seems able to catch the strain intensification in the weakest sections of samples and vertebrae.

Moreover, no damage law is included in the linear elastic numerical model. Indeed, the analyses are not aimed at the failure simulation itself, rather the focus is on the correlation between parameters estimated before damage manifestation, i.e. from linear elastic loading, and the beginning of the damage, which can be identified by the yielding. This choice is in accordance with [51]; also, it reduces the computational time and allows a quick run on computers installed on DXA systems.

Despite these drawbacks, the proposed methodology evidences potential clinical applications of the Strain Index of Bone, in particular on human lumbar vertebrae, that are the most stressed along the spine. The application of the procedure to humans is more challenging due to the complex shape of vertebrae, the intrinsic variability of the density, also due to the external cortical shells, and the presence of soft tissues, resulting in a wider greyscale for DXA images.

Table 2 compares *SIB*_*max*_ values with BMD (bone quantitative parameter accounting for density) and its T-score, and with TBS (bone qualitative parameter accounting for bone texture), commonly used to identify bone metabolic diseases such as osteoporosis. There is a consensual trend between T-score, TBS and *SIB*_*max*_. Indeed, as for BMD and T-score, we notice a remarkable difference between *SIB*_*max*_ values of the non-fractured patient (P_1_) and those of the fractured patients (the osteoporotic P_2_ and the osteopenic P_3_). Similarly, but less evident, TBS is the highest for P_1_, decreases for P_2_, and it is the lowest for P_3_.

The lumbar-fractured patient (P_2_), experiences the highest value of *SIB*_*max*_ at the lumbar vertebra L1, which is located in correspondence of the region where the spine changes its curvature and is statistically more prone to failure. The high *SIB*_*max*_ value could indicate that re-fracture is likely to occur, for this patient, in correspondence of vertebra L1. This consideration could not be drawn based only on BMD and T-score neither on TBS.

We can underline that BMD and T-score agree in the classification of patient P_1_ as a healthy condition of the spine, and of the patient P_2_ as an osteoporotic condition. On the other hand, P_3_ patient is an osteopenic patient according to T-score classification, i.e. between -1 and -2.5 [12]. For this patient, also *SIB*_*max*_ evidences average and peak values in between P_1_ and P_2_, but it seems interesting to note that the vertebra L1 of P_3_ experiences a quite high value of *SIB*_*max*_, in accordance with the lowest TBS. This observation could be an indicator of the local strain state in this particular vertebra. More generally, for patient P_3_, *SIB*_*max*_ values seem more in line with the values of P_2_ than of P_1_. All these comments could be helpful in the future to assume clinical decisions about specific therapy for metabolic bone diseases.

As a further comment, it seems that *SIB*_*max*_ accounts for the density information of BMD and T-score, as well as for the texture information of TBS, adding some details, as for L1 of P_2_.

Clearly, we only provided a few case studies to describe the possibilities of fracture prediction through the proposed numerical procedure. Further data need to be collected before fully assessing, with meaningful statistics, the ability of *SIB* to predict the event and the location of a failure. At present, DXA data are being processed for healthy patients, as a control group, and for osteoporotic patients, in order to support *SIB* predictions with statistical relevance. Data from periodic check-ups, combined with the temporal clinical histories of the patients, are also being cross-checked to support the capability of *SIB* to predict bone failures. In the future, this procedure, based on Strain Index of Bone measurements, could also support, together with BMD and TBS, the clinical prediction of fragility fractures and monitor osteoporosis treatments.

## Supporting information

S1 File (DOCX)

## Abbreviations

$\bar{G}$: mean greyscale value of the scanned DXA matrix
*A*_*FEM*_ (mm^2^): equivalent cross section of 2D finite element models of porcine specimen
*A*_*s*_ (mm^2^): cross section of porcine specimen
*BMD* (g/cm^2^): areal Bone Mineral Density
*E*_*0*_ (MPa): initial elastic modulus, experimentally determined for porcine specimens
*E*_*0v*_ (MPa): elastic modulus of human vertebra
*E*_*FEM*_ (MPa): elastic modulus numerically calculated by 2D finite element model
*E*_*i*_ (MPa): elastic modulus of the *i-th* finite element
*F* (N): compressive applied load
*G_i_*: greyscale level corresponding to *i-th* pixel
*h* (mm): height of the finite element model
*i (-)*: variable scanning rows and columns of the greyscale matrix, ranging from 1 to N
*j (-)*: variable scanning rows of the greyscale matrix of the processed region of the human vertebra, ranging from 1 to n
*k*: variable scanning integration points of the finite element model, ranging from 1 to 4N
*N (-)*: total number of pixels of the scanned DXA matrix, corresponding to the total number of finite elements of the 2D model
*n (-)*: total number of rows, based on DXA image
*r*–(mm): radius of the cylindrical specimen
*SIB (-)*: Strain Index of Bone
*SIB_max_ (-)*: maximum Strain Index of Bone, evaluated from Eq 7
*SIB_med_ (-)*: mean Strain Index of Bone, evaluated from Eq 6
*t** (mm): equivalent thickness of the 2D finite element model of porcine specimen
*TBS (-)*: Trabecular Bone Score
*u*_*y*_ (mm): vertical displacement of the upper surface of the sample, numerically calculated
*vBMD* (g/cm^3^): volumetric Bone Mineral Density
*W* (kg): patient’s total body weight
*w* (mm): local width of the human vertebra, based on DXA image
*ε_pmin_ (-)*: minimum (maximum absolute value) principal strain at the integration points
*ε_ULT_ (-)*: ultimate strain at failure, experimentally obtained for porcine specimens
*ε_Y_ (-)*: yield strain, experimentally obtained for porcine specimens from a 0.2% offset criterion
*ρ*_*app*_ (g/cm^3^): apparent bone density
*σ*_*ULT*_ (MPa): ultimate strength, experimentally obtained for porcine specimens
*σ*_*Y*_ (MPa): yield strength, experimentally obtained for porcine specimens from a 0.2% offset criterion
