# Supplementary material for: A new finite element based parameter to predict bone fracture
Source: PLoS One. 2019 Dec 5;14(12):e0225905. doi: 10.1371/journal.pone.0225905 (PMC6894848; doi:10.1371/journal.pone.0225905)
Supplement: S1 File — (DOCX) [file pone.0225905.s001.docx]

**Supporting information**

A new finite element based parameter to predict bone fracture

Chiara Colombo^1^, Flavia Libonati^1^, Luca Rinaudo^2^,

Martina Bellazzi^1^, Fabio Massimo Ulivieri^3*^, Laura Vergani^1^

^1^ Department of Mechanical Engineering, Politecnico di Milano, Milan, Italy

^2^ TECHNOLOGIC S.r.l. Hologic Italia, Torino, Italy

^3^ Fondazione IRCCS Cà Granda Ospedale Maggiore Policlinico, Nuclear Medicine-Bone Metabolic Unit, Milan, Italy

* Corresponding author

E-mail: ulivieri@gmail.com

1. **Experimental testing protocol on porcine trabecular bone samples**
   1. **Sample preparation**

Porcine trabecular specimens were cut from six vertebral columns taken from six pigs. The spines included six lumbar vertebrae, L1 to L6, except for three, where the L6 was missing. At least one specimen was obtained from each lumbar vertebra, leading to a total of 40 samples tested. For the statistical analyses, the duplicates were removed, as two samples extracted from the same vertebra cannot be considered independent from each other. This led to a total of 33 samples. Porcine lumbar spines from one-year old animals were provided from a local butcher and then stored at 18°C until the sample cutting and the experimental testing. Samples were drilled using a core drilling device (inner diameter of 16 mm and 40 mm of length) along the anatomical direction of the vertebral column. Then, the samples were transferred to a lathing machine to reduce them to cylinders with the diameter of 13.8 mm and height of 30 mm. During the drilling and turning, the specimens were kept wet by adding water. To reduce the edge effects and eliminate local damage effects, the ends of the bone samples were glued (3P Scotch-WeldTM EPXTM Adhesive DP490) in custom-made aluminium end caps (inner diameter 14 mm, outer diameter 20 mm and height 15 mm). The end caps covered 3 mm of the specimens on the parallel sides. To obtain perfectly parallel surfaces, both ends of bone specimens were smoothed using a circular blade saw. The bone samples and the aluminium tubes were defatted using acetone before gluing. A custom-made alignment tool was used to keep the bone and the end caps aligned within the direction of axial loading. Specimens were kept frozen at -18°C, and then rehydrated in saline solution (NaCl 0.9%) at 4°C for 12 h before mechanical testing.


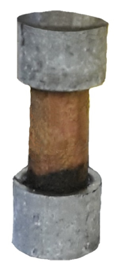


**S1 Fig – Bone sample with adhesively bonded aluminum end caps.**

- 1. **DXA scanning**

Porcine lumbar spines were first evaluated by means of a Hologic Discovery A system (Hologic Inc, Marlborough, Massachusetts, USA) installed at the Bone Metabolic Unit of the Nuclear Medicine of the Fondazione IRCCS Ca' Granda-Ospedale Maggiore Policlinico, Milan, Italy.

BMD, intended as areal bone mineral density (g/cm^2^) was assessed by the APEX software installed on the same machine. TBS was calculated automatically by software provided by Medimaps Group, Wilmington, US, and installed on the same machine. DXA image resolution was set to 0.5 mm, and each spine was placed in accordance to the correct anatomical planes. For each spine, after manually removing the residual ribs left from the butcher, we performed a lumbar scan, manually selecting the region of interest, to calculate TBS for each vertebra.

First, we performed the DXA scanning of each full spine, after removal from the animal, before the sample preparation, to make sure that the clinical parameters of the porcine spines were similar to those of adult human vertebrae, according to the International Society of Clinical Densitometry (https://www.iscd.org/ (April 2016)) and to the National Health and Nutrition Examination Survey (NHANES). Being the segmentation method in DXA scanning a manual procedure, we tested each vertebra three times to calculate the segmentation effect on our results.

We performed the same scans and analyses on the cylindrical samples before (i.e. pre-damage) and after (i.e. post-damage) mechanical testing. In each scanning, four cylindrical specimens were placed in the machine to perform the DXA scanning, keeping the orientation of the specimens similar to the complete vertebrae. Scanning lasted for about two minutes, and samples were kept frozen before and after scanning, to prevent any deterioration of the microstructure of the bone.

- 1. **Mechanical testing**

Monotonic compressive tests were performed in displacement control (strain rate of 0.0002 s-1; constant stroke rate of 0.05 mm/s). The initial length was determined by means of a caliper. The axial strain was measured using an extensometer (MTS 632.26F-20 with 8 mm gauge length) attached to the sample. Three preconditioning compression cycles up to 0.1% axial strain were performed, followed by monotonic loading until certain strain levels. After that, the specimens were unloaded and loaded three times until the same strain level, to obtain mechanical damage. Samples were divided in four groups, each set loaded until reaching a specific engineering strain value:

• Group G1%, with specimens loaded until 1% strain;

• Group G2%, with specimens loaded until 2% strain;

• Group G3.5%, with specimens loaded until 3.5% strain;

• Group G5%, with specimens loaded until 5% strain.

All the tests were conducted at room temperature. Data were acquired at a sampling rate of 20 Hz. The recording included time (s), stroke (S), force (F), and axial strain (ε). Normal stress (σ) was defined as the ratio of axial force (F) to primary area (A_0_), obtained from μCT scans for each sample. The initial elastic modulus (E_0_) was calculated using a moving regression with a box width of 0.2% strain to identify the stiffest section of the loading part. The yield stress (σ_Y_) and yield strain (ε_Y_) were obtained based on a 0.2% offset criterion. The ultimate stress (σ_ULT_) was attained as the maximum primary stress before densification and its corresponding strain as ultimate strain (ε_ULT_). Unloading stiffness (E) was calculated from the steepest part of the last loading cycle.

- 1. **Statistical analysis**

An unbalanced Latin Square design was used for mechanical testing. A statistical analysis was carried out in MATLAB® (R2015a) and SAS 9.2, and a p-value < 0.05 was assumed as the significant level. Clinical and mechanical parameters were analysed with simple linear regression models to find possible relationships.

ANCOVA was used to test the effect of group, location (fixed factors), and animal (random factor) on the difference of the considered variables between “before” and “after damage”, with the “before damage”-value as the covariate. Multiple pairwise comparisons between the group levels have been carried out by means of the Tukey’s HSD test with the p-value adjusted for multiplicity. In addition, a test of the null hypothesis that the difference between “before” and “after” damage is equal to zero has been carried out on the estimated least squares means of each group with a significance level of 0.0125, according to the Bonferroni’s correction.

1. **Details on *SIB_mean_* trend**

In addition to Figure 6.a of the manuscript, we add the information referred to *SIB_mean_*, of pre- and post- damaged porcine specimens.

**S2 Fig - Variation of: *SIB_mean_* before and after damage, for different mechanical damage levels (G1%, G2%, G3.5%, and G5%). The filled bars represent the results before damage and the unfilled ones after damage.**
